# Evaluating a screener to quantify PTSD risk using emergency care information: a proof of concept study

**DOI:** 10.1186/s12873-020-00308-z

**Published:** 2020-03-02

**Authors:** Willem F. van der Mei, Anna C. Barbano, Andrew Ratanatharathorn, Richard A. Bryant, Douglas L. Delahanty, Terri A. deRoon-Cassini, Betty S. Lai, Sarah R. Lowe, Yutaka J. Matsuoka, Miranda Olff, Wei Qi, Ulrich Schnyder, Soraya Seedat, Ronald C. Kessler, Karestan C. Koenen, Arieh Y. Shalev, International Consortium to Predict PTSD, Yael Errera-Ankri, Sarah Freedman, Jessie Frijling, Carel J. Goslings, Jan Luitse, Alexander McFarlane, Derrick Silove, Hanspeter Moergeli, Joanne Mouthaan, Daisuke Nishi, Meaghan O’Donnell, Mark Rusch, Marit Sijbrandij, Sharain Suliman, Mirjam van Zuiden

**Affiliations:** 1 Department of Population Health, New York University Langone Health, 227 E 30th St, New York, NY USA; 2 Department of Psychiatry, New York University School of Medicine, 1 Park Avenue, New York, NY 10016 USA; 3 Department of Epidemiology, Columbia University Mailman School of Public Health, 722 W. 168th St, New York, NY 10032 USA; 4 School of Psychology, University of New South Wales, Sydney, NSW 2052 Australia; 5 Department of Psychological Sciences, Kent State University, 144 Kent Hall, Kent, OH 44242 USA; 6 Department of Surgery, Medical College of Wisconsin, 9200 W. Wisconsin Avenue, Milwaukee, WI 53226 USA; 7 Department of Counselling, Developmental, and Educational Psychology, Lynch School of Education and Human Development, Boston College, Campion Hall Room 313, 140 Commonwealth Avenue, Chestnut Hill, MA 02467 USA; 8 Department of Psychology, Montclair State University, 1 Normal Avenue, Montclair, NJ 07043 USA; 9 Division of Health Care Research, Center for Public Health Sciences, National Cancer Center Japan, 5-1-1 Tsukiji, Chou-ku, Tokyo, 104-0045 Japan; 10 Department of Psychiatry, Academic Medical Center, University of Amsterdam, Meibergdreef 9, 1105 AZ Amsterdam, The Netherlands; 11 Arq Psychotrauma Expert Group, Postbus 240, 1110 AE Diemen, The Netherlands; 12 Department of Psychiatry and Psychotherapy, University Hospital Zurich, PO Box 1931, Lenggstrasse 31, 8032 Zürich, Switzerland; 13 Department of Psychiatry, Stellenbosch University, Private Bag X1, Matieland, Stellenbosch, 7602 South Africa; 14 Department of Health Care Policy, Harvard Medical School, 180 Longwood Avenue, Boston, MA 02115 USA; 15 Department of Epidemiology, Harvard T.H. Chan School of Public Health, Kresge 505, 677 Huntington Avenue, Kresge Building, Boston, MA 02115 USA

**Keywords:** Emergency care admissions, Post-traumatic stress disorder, Peritraumatic symptoms, Sequential prediction, Mega-analysis

## Abstract

**Background:**

Previous work has indicated that post-traumatic stress disorder (PTSD) symptoms, measured by the Clinician-Administered PTSD Scale (CAPS) within 60 days of trauma exposure, can reliably produce likelihood estimates of chronic PTSD among trauma survivors admitted to acute care centers. Administering the CAPS is burdensome, requires skilled professionals, and relies on symptoms that are not fully expressed upon acute care admission. Predicting chronic PTSD from peritraumatic responses, which are obtainable upon acute care admission, has yielded conflicting results, hence the rationale for a stepwise screening-and-prediction practice. This work explores the ability of peritraumatic responses to produce risk likelihood estimates of early CAPS-based PTSD symptoms indicative of chronic PTSD risk. It specifically evaluates the Peritraumatic Dissociative Experiences Questionnaire (PDEQ) as a risk-likelihood estimator.

**Methods:**

We used individual participant data (IPD) from five acute care studies that used both the PDEQ and the CAPS (*n* = 647). Logistic regression calculated the probability of having CAPS scores ≥ 40 between 30 and 60 days after trauma exposure across the range of initial PDEQ scores, and evaluated the added contribution of age, sex, trauma type, and prior trauma exposure. Brier scores, area under the receiver-operating characteristic curve (AUC), and the mean slope of the calibration line evaluated the accuracy and precision of the predicted probabilities.

**Results:**

Twenty percent of the sample had CAPS ≥ 40. PDEQ severity significantly predicted having CAPS ≥ 40 symptoms (*p* < 0.001). Incremental PDEQ scores produced a reliable estimator of CAPS ≥ 40 likelihood. An individual risk estimation tool incorporating PDEQ and other significant risk indicators is provided.

**Conclusion:**

Peritraumatic reactions, measured here by the PDEQ, can reliably quantify the likelihood of acute PTSD symptoms predictive of chronic PTSD and requiring clinical attention. Using them as a screener in a stepwise chronic PTSD prediction strategy may reduce the burden of later CAPS-based assessments. Other peritraumatic metrics may perform similarly and their use requires similar validation.

**Trial registration:**

Jerusalem Trauma Outreach and Prevention Study (J-TOPS): NCT00146900.

## Background

Traumatic injury is common in the general population [1]. Worldwide, 56.2 million individuals sustain injuries that require hospital admission per year [2]. In the United States alone, emergency departments (EDs) treat 39 million injury survivors per year, comprising 28% of all annual ED visits [3]. Traumatic injury can be a significant precipitating event for developing posttraumatic stress disorder (PTSD; e.g. [4–7],), which, in its chronic form, is tenacious and debilitating [8, 9], and which early cognitive behavioral interventions may efficiently mitigate [10–13].

ED admissions provide crucial points of contact with patients at risk of developing PTSD. The best-known metrics for estimating chronic PTSD risk are early PTSD symptoms, as assessed by structured clinical interviews such as the Clinician-Administered PTSD Scale (CAPS [14, 15];). However, most PTSD symptoms (e.g., insomnia, avoidance, social withdrawal) are not fully expressed during ED admission, and their reliable assessment using the CAPS requires two to 4 weeks of symptom duration [16]. Using the CAPS is additionally burdensome because it requires time, clinical expertise, and re-contacting patients. For example, a large outreach and prevention study that inclusively screened 5000 consecutive ED admissions and clinically evaluated 750 deemed at risk for PTSD within a month of trauma exposure [17] found that bringing one survivor with acute PTSD to treatment required 6.09 h of structured telephone interviews and 5.09 h of clinical, CAPS-based assessments.

Alternatively, the immediate reactions to traumatic events, otherwise known as *peritraumatic responses*, are fully expressed and measurable upon ED admission. Unfortunately, measures of peritraumatic distress perform rather poorly as predictors of chronic PTSD [18–20].

Peritraumatic responses may, however, better predict early PTSD symptoms [21], previously shown to robustly predict chronic PTSD [14]. As such, peritraumatic responses might be used to identify a subset of trauma survivors likely to develop early PTSD symptoms indicative of high chronic PTSD risk. This type of stepwise strategy can reduce the burden of unselective early clinical assessment. Similar stepwise screening-and-assessment approaches regularly inform disease prevention in other medical fields (e.g., mammography towards breast biopsy, stool blood towards colonoscopy, effort test towards angiography).

Several short, self-administered instruments have been used to assess peritraumatic reactions, such as the Peritraumatic Distress Inventory (PDI, a 13-item instrument [22];) and the Peritraumatic Dissociative Experiences Questionnaire (PDEQ, a 10-item instrument [23];). The latter evaluates the occurrence, during or shortly after trauma exposure, of dissociation symptoms (discontinuity or disintegration of conscious awareness [24]) previously associated with PTSD risk.

A prior study by our group used multinational longitudinal data [14] to evaluate the efficiency of CAPS scores obtained within 60 days of a traumatic event as an early risk indicator of chronic PTSD. Within that work, an initial CAPS score greater than 40 points (CAPS ≥ 40) was associated with higher than average likelihood (> 11%) of developing chronic PTSD. Extending upon these results, this study evaluates the efficiency of the PDEQ as an estimator of the likelihood of expressing CAPS ≥ 40 30 to 60 days after ED admission. As in our previous study, we pooled together eligible studies’ item-level individual data and opted to use a continuous risk-estimate approach, i.e., produce probability scores of CAPS ≥ 40 for each incremental PDEQ score. We thereby deviated from the more frequently used case classification approach, which produces a threshold (cut-off score) of the predictor (herein the PDEQ) that optimally classifies survivors into those with or without the outcome of interest (herein CAPS ≥ 40). Derived by a logistic regression, our risk estimate approach additionally allowed us to incorporate the effect of several known PTSD risk indicators (e.g., sex, prior trauma exposure) in the model.

This work constitutes a *mega-analysis,* in which raw data from participating studies are analyzed at the *individual level,* rather than at the study-level, as in a meta-analysis. After controlling for contributing studies’ heterogeneities, as detailed below, this approach allows the pooled data to be used as a larger, more diverse study sample.

Data for this work were drawn from the same ED-based, multinational dataset of trauma survivors [25], wherein the PDEQ had the widest availability of all peritraumatic measures. Participants were traumatic injury survivors treated in EDs in the United States, the Netherlands, and Israel. Peritraumatic dissociation was measured shortly after ED treatments. CAPS symptoms were measured 30 to 60 days after ED treatments.

## Methods

### Sources of data

Data for this work were obtained from the pooled dataset of the International Consortium to Predict PTSD (ICPP). Studies contributing to the ICPP had longitudinally evaluated adult civilians who were treated in general hospital EDs following traumatic injuries. Mechanisms of injury included motor vehicle accidents, other non-interpersonal accidents (e.g., falls, burns), and interpersonal harm (e.g., assault, sexual violence). Table 1 presents participating individual studies’ characteristics. Extended information on the ICPP’s design and data harmonization can be found in Qi et al. (2018) [25].

**Table 1:** Participating Studies’ Designs, Countries of Origin, Sample Sizes, and Sample Characteristics

Study	Design	Country	Studies’ total samples	Current Eligible participants^a^	Trauma Types among participants (MVA, OA, IV)^b^
Irish et al., 2008 [26]	Observation	USA	406	144	100, 0, 0%
Mouthaan et al., 2013 [27]	Observation	The Netherlands	852	250	62.00, 34.80, 3.20%
Shalev et al., 2000 [28]	Observation	Israel	235	123	86.18, 6.50, 7.32%
Shalev et al., 2008 [29]	Observation	Israel	279	88	80.23, 4.65, 15.12%
van Zuiden et al., 2017 [30]	Intervention	The Netherlands	54	42	69.05, 21.43, 9.52%

^a^Reflects those meeting inclusion criteria specified under method/participants

^b^*MVA* motor vehicle accident, *OA* other accident, *IV* interpersonal violence

### Participants

Participants for this work were those included in five ICPP studies [26–30] that sequentially administered the PDEQ [23] and the Clinician-Administered PTSD Scale for DSM-IV (CAPS [15]); Table 1). Eligible studies’ participants (*n* = 647; Table 2) were included if they had a PDEQ assessment within 30 days of trauma exposure and a CAPS interview between 30 and 60 days after trauma exposure. Participants included in this work did differ from those not included (*n* = 1179) on age (*p* = 0.045, *t*_1048_ = 2.00), but neither on sex (*p* = 0.334), PDEQ severity score (*p* = 0.179, *t*_1052_ = − 1.34), or prior trauma history (*p* = 0.334). However, after application of the Hoch-Bonferroni adjustment for multiple comparisons, the *p*-value for age became non-significant (*p* = 0.179). Confidence intervals are supplied to complement the statistical tests.

**Table 2:** Sample demographics by CAPS score at 30–60 days post-trauma

Variable	CAPS IV < 40	CAPS IV ≥ 40	Test Statistic	p	Total Sample
Participants (N (%))	517 (79.91%)	130 (20.09%)			647
PDEQ Total Score (Mean; (SD); [95%CI])	15.15 (6.07) = [14.62, 15.67]	21.79 (6.53) [20.66, 22.93]	−10.51 *df* = 189	0.001^a^	16.48 (6.71) [15.97, 17.00]
Age (Mean; (SD); [95%CI])	38.91 (15.80) [37.55, 40.28]	34.49 (13.02) [32.23, 36.75]	3.31 *df* = 234	0.001^a^	38.02 (15.38) [36.84, 39.21]
Sex			N/A	0.001^b^	
Female [N(%)]	208 (40.23%)	75 (57.69%)			283 (43.74%)
Male [N(%)]	309 (59.77%)	55 (42.31%)			
Prior trauma ^c^			N/A	0.002 ^b^	
None [N (%)]	62 (12.23%)	10 (7.75%)			72 (11.32%)
Non-Interpersonal [N (%)]	208 (41.03%)	36 (27.91%)			244 (38.36%)
Interpersonal [N (%)]	237 (46.75%)	83 (64.34%)			320 (50.31%)
Current Trauma ^d^			N/A	0.001 ^b^	
MVA [N(%)]	400 (77.52%)	103 (79.84%)			503 (77.98%)
Non-MVA [N(%)]	97 (18.80%)	11 (8.53%)			108 (16.74%)
Interpersonal violence [N(%)]	19 (3.68%)	15 (11.63%)			34 (5.27%)

*Abbreviations*: *CAPS* clinician administered PTSD Scale for DSM IV, *PDEQ* peri-traumatic dissociation questionnaire, *MVA* motor vehicle accidents, *df* degrees of freedom, *N/A* not applicable

^a^ Welch’s *t*-test

^b^ Fisher’s test

^c^ Data on prior trauma history were missing for 12 participants

^d^ Data on current trauma were missing for 2 participants

### Instruments

Because they were performed before the publication of DSM-5, ICPP studies used the DSM-IV version of the CAPS. The CAPS is a structured clinical interview that evaluates the intensity and the frequency of 17 DSM-IV PTSD symptom criteria on a scale of 0–4 (score range = 0–136). The CAPS was administered by trained clinicians 30 to 60 days after ED treatment, a time bracket within which a DSM-IV diagnosis of Acute PTSD is warranted. The CAPS has demonstrated excellent psychometric properties, with interrater reliability for continuous CAPS scores consistently exceeding .90 [31], and similarly high test-retest reliability. Alpha scores for the CAPS range from .80 to .90 across symptom clusters and the disorder as a whole [31]. In our data, Cronbach’s alpha for the intrusion, avoidance, and hyperarousal symptom clusters were 0.91, 0.87, and 0.87, respectively. Additionally, Cronbach’s alpha was 0.94 for the 17 CAPS items together.

The PDEQ is a self-report questionnaire measuring peritraumatic dissociation [23]. The instrument’s 10 items are rated on a 5-point scale of 1 (“not at all”) to 5 (“extremely true”). Seven PDEQ items were consistently used across the five participating studies: “Blanked out”, “Being on automatic pilot”, “Sense of time changed”, “What happened seemed unreal”, “Floating above the scene”, “Feeling disconnected from the body”, and “Things happened without awareness”, yielding a 7–35 point score range. The 10-item and revised 8-item PDEQ have both demonstrated good internal consistency, with alpha scores ranging from .81 to .85 [22, 32], as well as good test-retest reliability (*r* = .85, *p* < .01) on the 8-item PDEQ [32]. In our sample, Cronbach’s alpha for the PDEQ was 0.80.

Additionally, *sex, age, current trauma type* (motor vehicle accident, other accident, or interpersonal violence), and *lifetime trauma history* (no prior trauma, prior non-interpersonal trauma, and prior interpersonal trauma) were evaluated.

### Data analysis

#### Main outcome measure

The categorical outcome measure for this work was a CAPS total score ≥ 40 points, reflective of moderate PTSD severity and previously associated with ≥11% likelihood of PTSD 9 to 15 months after trauma exposure [14].

To compare participants with CAPS ≥ 40 scores with those below that threshold, we used Welch’s *t*-test for continuous variables and Fisher’s test for categorical variables. A logistic regression analysis assessed the impact of covariates on the association between PDEQ total score and CAPS score ≥ 40. Complete-case analysis was used in instances of missing data.

To assess whether pooling of individuals from different study sites was appropriate for this analysis, the I^2^, a measure of unexplained heterogeneity between effect sizes, assessed heterogeneity of study-specific log odds ratios derived from logistic regression in study-stratified models. As an additional measure of heterogeneity, a test of the homogeneity of the effect sizes was conducted via the Q statistic.

*Logistic regression* was used to derive the probability of a CAPS score ≥ 40 given the PDEQ total score. Following Debray et al. (2013) [33], both stacked models and stratified intercept models were derived, where a stacked intercept model pools individuals without accounting for data source, and a stratified intercept model includes a term for data source. A random effects model was not used due to the low number of studies.

The significance of logistic regression coefficients was tested via Z-test at the 5% level of significance. Predicted probabilities were calculated through conversion of the predicted log odds of the outcome, and confidence intervals were calculated.

The accuracy of the predicted probabilities was assessed using the Brier score, which is a number between 0 and 1, with a lower score indicating a stronger predictive model [34]. Additionally, the Brier Skill Score was also used, which ranges from 0 to 1, with a number closer to 1 indicating a stronger predictive model [34]. The Brier Skill Score calculates the model’s improvement in Brier Score relative to a naïve model. The area under the receiver-operating characteristic curve (AUC) was calculated to assess the ability of the PDEQ to discriminate between cases (CAPS ≥ 40) and non-cases by using the predicted probabilities from the model compared to the observed outcomes. Calibration slope was calculated through bootstrap resampling of the study with 1000 repetitions to measure the accuracy of the predicted probabilities, with a slope closer to 1 indicating more accurate predicted probabilities and slopes greater than 1 reflecting underfitting of probabilities and slopes less than 1 representing overfitting of predicted probabilities. For the calibration slope calculation, observed outcomes were regressed on predicted logits for each iteration of the bootstrap via logistic regression and the beta for the predicted logits averaged across repetitions. Ridge penalizations and transformations of PDEQ score were considered, if underfitting and overfitting were found by examining calibration slope and plots.

The strength of the predicted probabilities and the discriminatory power of the final model were further assessed in each study separately in order to assess the validity of the model in different samples by calculating the AUC and Brier Skill Score for each study. Additionally, to assess the increase in predictive power by inclusion of the PDEQ relative to demographic and trauma variables alone, DeLong’s test was performed between the adjusted PDEQ model and the demographic and trauma variable alone model [35].

### Sensitivity analyses

The effect of PDEQ assessment timing (number of days from ED treatment) on the association between PDEQ and CAPS ≥ 40 score was evaluated via the incorporation of an interaction term between PDEQ score and the days since trauma for the PDEQ assessment. Likewise, the effect of CAPS assessment timing (days from ED treatment) on the association between PDEQ and CAPS ≥ 40 was evaluated via the incorporation of an interaction term between PDEQ score and the days since trauma of the CAPS assessment. A model where the first CAPS assessment was used, regardless of date, to include more of the sample was called the unrestricted model.

All significance tests were at the 5% level of significance, and all confidence intervals were at the 95% level of confidence. All analyses were conducted with R version 3.4.0 [36].

## Results

### Descriptive statistics

Table 2 presents the sample characteristics. There were 647 subjects from 5 studies. One hundred and thirty participants (20.09%; subsequently referred to as “cases”) had a CAPS ≥ 40 in the 30–60 days after trauma time period. Cases had significantly higher PDEQ scores than non-cases (*p* < 0.001, *t*_189_ = − 10.51) and were younger (*p* = 0.001, *t*_234_ = 3.31). Cases were more likely to have interpersonal trauma as their most recent trauma (*p* < 0.001) and have experienced prior interpersonal trauma (*p* = 0.002). Women had 2.02 (36.05% vs 17.79, 95% CI = [1.35, 3.05]) times the odds of having a CAPS score over 40 than men (*p* < 0.001). Eleven participants were missing data on prior trauma history and two participants were missing data on current trauma type. Confidence intervals are supplied as an alternative means of assessing group differences.

### Assessing study-dependent heterogeneity and appropriateness of pooling

**Fig. 1:**
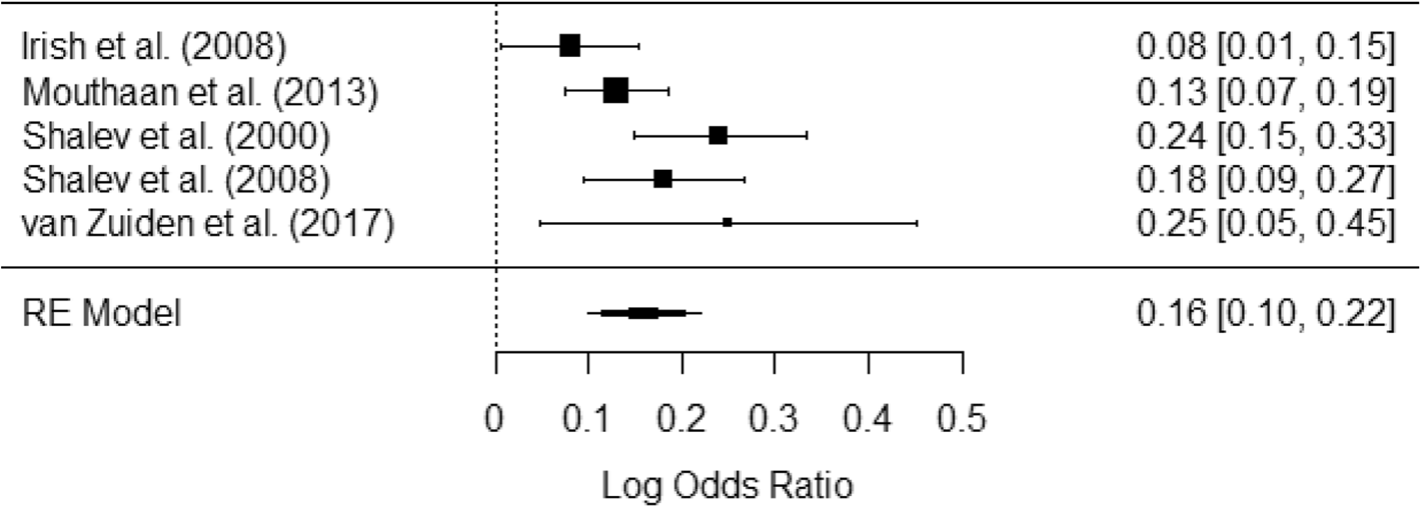
Forest plot of log odds ratios between PDEQ Score and CAPS ≥ 40 for individual studies

The I^2^ was 56.67%, indicating moderate heterogeneity in regression coefficients. Q Test statistic for heterogeneity was 8.92 with 4 degrees of freedom (*p* = 0.063), which suggests that there is insufficient evidence to suggest that effect sizes are heterogeneous in a manner that is statistically significant. Odds measuring the relative increase in log odds of having a CAPS score over 40 for a one unit increase in PDEQ score and 95% confidence intervals for each of the studies were as follows (studies’ references in parentheses): *b* = 0.08 [0.01, 0.16] [26], *b* = 0.13 [0.08, 0.19] [27], *b* = 0.24 [0.15, 0.33] [28], *b* = 0.18 [0.09, 0.27] [29], and *b* = 0.25 [0.05, 0.46] [30]. Due to the moderate amount of heterogeneity between studies, pooling the studies for analysis was deemed appropriate. See Fig. 1.

### Prediction of CAPS score over 40 by PDEQ – comparing models (Table 3)

All models are without transformations and ridge penalizations.

**Table 3:** Logistic Regression Coefficients and Model Fit Statistics

Variable	Stacked Model- PDEQ Only^1^	Stacked Model-Adjusted^2^	Stratified Model-PDEQ Only^3^	Stratified Model- Adjusted^4^	Unrestricted Model- PDEQ Only^5^	Unrestricted Model – Adjusted^6^	Stacked Model – Demographics Only^7^	Stratified Model – Demographics Only^8^
Intercept	−4.22 (0.35)*	−4.64 (0.62)*	− 4.32 (0.48)*	−4.90 (0.68)*	− 3.99 (0.29)*	− 4.53 (0.52)*	−1.79 (0.44)*	−1.80 (0.49)*
PDEQ	0.15 (0.02)*	0.16 (0.02)*	0.16 (0.02)*	0.16 (0.02)*	0.13 (0.01)*	0.13 (0.01)*		
Age		−0.02 (0.008)		−0.01 (0.01)		− 0.01 (0.01)	−0.02 (0.007)*	− 0.01 (0.008)
Sex^9^		0.53 (0.23)*		0.52 (0.23)*		0.44 (0.19)	0.74 (0.21)*	0.72 (0.21)*
Current Trauma								
MVA		Reference:		Reference		Reference	Reference	Reference
Non-interpersonal		−0.83 (0.39)*		−0.66 (0.40)		−0.53 (0.32)	−0.55 (0.35)	− 0.39 (0.37)*
Interpersonal		0.80 (0.42)		0.86 (0.43)*		0.87 (0.34)*	1.21 (0.38)*	1.15 (0.40)*
Prior trauma								
No prior trauma		Reference		Reference		Reference	Reference	Reference
Non-interpersonal		0.37 (0.44)		0.34 (0.45)		0.63 (0.37)	0.30 (0.40)	0.29 (0.41)
Interpersonal		1.01 (0.42)*		0.95 (0.43)*		1.03 (0.35)*	0.96 (0.38)*	0.90 (0.39)*
Data Source								
Shalev et al., 2008 [29]			Reference	Reference				Reference
Shalev et al., 2000 [28]			0.66 (0.36)	0.37 (0.38)				0.16 (0.34)
Irish et al., 2008 [26]			0.22 (0.37)	0.27 (0.39)				−0.20 (0.36)
van Zuiden et al., 2017 [30]			−0.44 (0.35)	−0.23 (0.39)				−0.55 (0.35)
Mouthaan et al., 2014 [37]			0.08 (0.47)	0.04 (0.51)				−0.07 (0.47)
Model Fit Statistics								
AUC	0.77	0.81	0.78	0.81	0.75	0.78	0.69	0.70
Brier Score	0.13	0.13	0.13	0.13	0.13	0.13	0.15	0.15
Brier Skill Score	0.17	0.22	0.20	0.22	0.12	0.15	0.08	0.09
Calibration Slope	1.007	0.941	0.966	0.908	1.004	0.946	0.903	0.836

* *p* < 0.05. All regression coefficients are reported as *b* (SE)

*Abbreviations*: *PDEQ* peri-traumatic dissociative experiences questionnaire, *MVA* motor vehicle accident, *AUC* area under the receiver-operating curve

^1^ Stacked model with PDEQ only

^2^ Stacked model adjusted for age, gender, trauma type, and prior trauma

^3^ Stratified model with PDEQ only

^4^ Stratified model adjusted for age, gender, trauma type, and prior trauma

^5^ Unrestricted model with PDEQ only

^6^ Unrestricted model adjusted for age, gender, trauma type, and prior trauma

^7^ Stacked model with demographic variables (i.e., age, sex, mechanism of injury, prior trauma history) only

^8^ Stratified model with demographic variables (i.e., age, sex, mechanism of injury, prior trauma history) only

^9^ Male is the reference group

The stacked model with PDEQ only (Table 3, column 2) had an intercept of − 4.22 and a PDEQ coefficient of 0.15 (SE = 0.02, *p* < 0.001), meaning that a one unit increase in PDEQ score corresponds to a 1.17 (95% CI = [1.13, 1.21]) fold increase in the odds of having a CAPS score over 40.

The stacked model adjusted for age, sex, trauma type, and prior trauma history (Table 3, column 3): The model intercept was − 4.64, and the coefficient for the PDEQ was 0.16 (SE = 0.02, *p* < 0.001), meaning that a one unit increase in PDEQ score corresponds to a 1.17 (Beta = 0.02, 95% CI = [1.13, 1.22]) fold increase in the risk of having a CAPS score over 40. Additionally, if the trauma was a non-interpersonal, non-motor vehicle accident, the odds of having a CAPS score over 40 were reduced by 57% relative to motor vehicle accidents (Beta = − 0.83, OR = 0.43, 95% CI = [0.20, 0.93], *p* = 0.031). Also, prior interpersonal trauma was significant in increasing the odds of having a CAPS score over 40 by a factor of 2.75 relative to no prior trauma (Beta = 1.01, 95% CI = [1.21, 6.28], *p* = 0.016). Women were found to have odds of having a CAPS score over 40 that were 70% greater than men (Beta = 0.53, OR = 1.70, 95% CI = [1.09, 2.67], *p* = 0.019). All other variables were statistically non-significant (*p* > 0.05).

The stratified model with PDEQ only (Table 3, column 4) had an intercept of − 4.32 and a PDEQ coefficient of 0.16 (SE = 0.02, p < 0.001), meaning that a one-unit increase in PDEQ score corresponds with a 1.17 (95% CI = [1.13, 1.21]) fold increase in risk of having a CAPS score over 40, when accounting for study-source.

The stratified model adjusted for age, sex, trauma type, and prior trauma history (Table 3, column 5) had an intercept of − 4.90, and the coefficient for the PDEQ was 0.16 (SE = 0.02, p < 0.001), meaning that a one-unit increase in PDEQ score corresponds to a 1.17 (95% CI = [1.13, 1.22]) fold increase in the risk of having a CAPS score over 40. Prior interpersonal trauma was associated with an increase in the risk of having a CAPS score over 40 by a factor of 2.58 relative to no prior trauma (Beta = 0.95, 95% CI = [1.11, 5.99], *p* = 0.027). Additionally, if the trauma was an incident of interpersonal violence, the odds of having a CAPS score over 40 were increased by a factor of 2.36 relative to motor vehicle accidents (Beta = 0.86, 95% CI = [1.01, 5.51], *p* = 0.048). Lastly, women had 1.68 times the odds of having a CAPS score over 40 (Beta = 0.52, 95% CI = [1.07, 2.64], *p* = 0.024). Neither age nor any of the data sources were statistically significant (p > 0.05). See Table 3.

### Final model selection and validation

Based on AUC and Brier Skill Score, the stacked and adjusted model performed better than other models. Brier Skill Scores ranged from 0.08 to 0.31. AUCs ranged from 0.73 to 0.85. The validation results indicate fair to good discriminatory power, according to AUC, and weak to moderate improvements in predicted probabilities relative to a naïve model, according to Brier Skill Score. Table 3 additionally shows a model featuring only demographic and trauma variables. The stacked and adjusted PDEQ model has a higher AUC (0.81) than the demographic and trauma variable alone model (0.69). DeLong’s test illustrates that the stacked and adjusted PDEQ model has a statistically significantly different AUC than the demographic and trauma variable only model (Z = − 4.94, *p* < 0.001).

### Likelihood estimate calculation

CAPS ≥ 40 (high risk) likelihood estimates based on the stacked and adjusted model are provided as a web-based risk calculator on https://wvdmei.shinyapps.io/results_lookup/. This calculator reports and visualizes individual acute PTSD likelihood estimates based on either the PDEQ alone, or the PDEQ plus sex, age, mechanism of injury, and lifetime trauma exposure. Table 4 provides predicted probabilities and 95% confidence intervals for different covariate values of the regression model stacked and adjusted for covariates.

**Table 4:** Predicted probabilities of PTSD at 30–60 days post-trauma based on PDEQ score, sex, mechanism of injury, and lifetime trauma history

Sex:	Male									Female								
Current trauma:	Motor Vehicle Accidents			Interpersonal Trauma			Non-Interpersonal Trauma^a^			Motor Vehicle Accidents			Interpersonal Trauma			Non-Interpersonal Trauma^a^		
Past trauma:	None	Non-Interpersonal	Interpersonal	None	Non-Interpersonal	Interpersonal	None	Non-Interpersonal	Interpersonal	None	Non-Interpersonal	Interpersonal	None	Non-Interpersonal	Interpersonal	None	Non-Interpersonal	Interpersonal
PDEQ																		
7	1.60 [0.62, .410]	2.29 [1.19, 4.37]	4.29 [2.45, 7.41]	3.49 [1.14, 10.23]	4.95 [1.84, 12.67]	9.05 [3.72, 20.42]	0.70 [0.22, 2.26]	1.01 [0.38, 2.64]	1.91 [0.77, 4.63]	2.70 [1.05, 6.79]	3.85 [2.04, 7.13]	7.09 [4.09, 12.04]	5.81 [1.85, 16.81]	8.16 [2.99, 20.41]	14.51 [5.89, 31.50]	1.19 [0.36, 3.87]	1.71 [0.64, 4.49]	3.21 [1.27, 7.85]
10	2.56 [1.04, 6.14]	3.64 [2.04, 6.43]	6.73 [4.18, 10.68]	5.51 [1.90, 14.91]	7.75 [3.07, 18.21]	13.82 [6.14, 28.23]	1.13 [0.36, 3.42]	1.62 [0.65, 3.96]	3.04 [1.31, 6.88]	4.28 [1.77, 10.01]	6.06 [3.49, 10.29]	10.96 [6.92, 16.91]	9.04 [3.08, 23.70]	12.53 [4.96, 28.22]	21.47 [9.59, 41.35]	1.91 [0.61, 5.80]	2.72 [1.09, 6.66]	5.07 [2.16, 11.46]
15	5.49 [2.41, 12.02]	7.73 [4.77, 12.27]	13.78 [9.56, 19.47]	11.44 [4.35, 26.83]	15.69 [6.92, 31.76]	26.21 [13.3, 45.14]	2.46 [0.86, 6.86]	3.51 [1.53, 7.83]	6.49 [3.07, 13.19]	9.02 [4.08, 18.77]	12.49 [8.11, 18.76]	21.42 [15.42, 28.95]	18.05 [6.95, 39.36]	24.08 [10.93, 45.06]	37.72 [19.94, 59.56]	4.13 [1.43, 11.32]	5.84 [2.56, 12.76]	10.58 [5.01, 21.00]
20	11.41 [5.31, 22.82]	15.65 [10.24, 23.17]	26.15 [19.31, 34.39]	22.24 [9.38, 44.16]	29.19 [14.44, 50.17]	44.04 [25.75, 64.10]	5.29 [1.95, 13.6]	7.45 [3.46, 15.33]	13.33 [6.78, 24.53]	18.00 [8.83, 33.22]	24.03 [16.88, 32.99]	37.65 [29.45, 46.62]	32.78 [14.56, 58.26]	41.27 [21.83, 63.87]	57.29 [36.11, 76.11]	8.70 [3.23, 21.38]	12.07 [5.73, 23.69]	20.77 [10.83, 36.15]
25	22.19 [10.89, 39.97]	29.12 [19.56, 40.98]	43.96 [33.34, 55.16]	38.79 [18.57, 63.78]	47.72 [26.96, 69.31]	63.54 [42.96, 80.13]	11.02 [4.23, 25.76]	15.14 [7.34, 28.65]	25.41 [13.78, 42.06]	32.71 [17.49, 52.72]	41.19 [30.31, 53.01]	57.22 [46.75, 67.08]	51.93 [27.38, 75.58]	60.88 [38.03, 79.79]	74.82 [55.24, 87.74]	17.43 [6.94, 37.40]	23.32 [11.90, 40.67]	36.74 [21.15, 55.70]
30	38.71 [20.41, 60.87]	47.65 [32.93, 62.78]	63.47 [49.72, 75.33]	58.39 [32.73, 80.19]	66.91 [43.79, 84.00]	79.43 [61.18, 90.44]	21.52 [8.7, 44.13]	28.33 [14.42, 48.11]	43.00 [25.19, 62.83]	51.85 [30.88, 72.19]	60.81 [46.74, 73.29]	74.76 [63.48, 83.47]	70.53 [44.70, 87.63]	77.52 [56.57, 90.12]	86.81 [72.22, 94.34]	31.86 [13.90, 57.53]	40.26 [22.38, 61.16]	56.26 [36.24, 74.44]
35	58.32 [34.30, 78.95]	66.84 [48.95, 80.91]	79.38 [65.50, 88.64]	75.66 [50.23, 90.54]	81.75 [61.48, 92.63]	89.53 [76.17, 95.81]	37.79 [16.62, 64.94]	46.68 [25.70, 68.90]	62.56 [40.65, 80.31]	70.46 [47.68, 86.20]	77.46 [63.08, 87.36]	86.78 [76.94, 92.81]	84.13 [62.76, 94.34]	88.42 [72.84, 95.60]	93.58 [84.15, 97.56]	50.88 [25.33, 75.98]	59.88 [37.27, 78.94]	74.02 [53.75, 87.48]

^a^Excludes motor vehicle accidents. Average age for the sample (38.0) was used for this reference table. For more information on the influence of age, visit https://wvdmei.shinyapps.io/results_lookup/

### Sensitivity analyses

#### Influence of assessment timing

The median time from ED treatment to the first PDEQ administration was 9 days (25th percentile = 3.50, 75th percentile = 17.50, Range = 0–30). Median time to the first CAPS administration between 30 to 60 days was 42 days (25th percentile = 35, 75th percentile = 49.00, Range = 31–60). There was insufficient evidence to suggest that timing of the PDEQ moderated the relationship between PDEQ score and a CAPS ≥ 40 (*b* = − 0.001, SE = 0.002, *p* = 0.610) based on a test of the interaction term. However, the data did suggest that later timing of the CAPS assessment moderated the relationship between PDEQ score and CAPS ≥ 40 by decreasing the size of the odds ratio for PDEQ score (*b* = − 0.01, SE = − 0.004, *p* = 0.019), via a test of the interaction term.

## Discussion

Our results demonstrate that peritraumatic reactions, evaluated here by the PDEQ, can be used to produce a risk estimate of acute PTSD (CAPS ≥ 40) symptoms indicative of above-average risk of chronic PTSD. The risk model was shown to have fair-to-good performance when validated in the individual studies, thereby exhibiting utility in heterogeneous populations. Importantly, the CAPS ≥ 40 subgroup concerned about one fifth (20.09%) of the total sample, illustrating the ability of a peritraumatic screening to significantly reduce the number of structured clinical evaluations (i.e., CAPS assessments) administered within 30 to 60 days required to quantify survivors’ risk of chronic PTSD. The model used in this work additionally documented the effect of several covariates (sex, age, trauma type and lifetime trauma exposure) on PDEQ-based risk estimation.

Extending previous longitudinal studies that used structured clinical interviews to predict downstream PTSD [14, 38], our results demonstrate the utility of using a short self-report measure to produce a likelihood estimate of early CAPS scores, themselves predictive of chronic PTSD. Self-report measures, such as the PDEQ, may be useful in ED settings given their minimal burden on personnel and resources. They therefore present plausible candidates for a stepwise, screen-and-assess approach to PTSD risk assessment, capable of guiding early prevention. In this work specifically, the 7-item PDEQ represents a particularly short screening tool that could be well-suited for time-sensitive ED assessment.

This model introduces to ED psychiatry a nomogram approach extensively used in other areas of medicine, allowing physicians to assign a risk likelihood percentage to each person at risk given a set of predictors. The model also innovatively combines several risk factors in its individual likelihood estimates, demonstrating the powerful effect of quickly measurable covariates on PTSD risk.

In this work, given the multiplicity of predictors and individual risk estimates, we do not recommend an a priori cut-off score for follow-up assessment. Within this approach, clinicians and service providers have the flexibility to determine follow-up care based on hospital resources and patient-practitioner clinical decision-making. Likewise, the predictive models with and without covariates allow emergency personnel to use either a longer (but more illustrative) or more efficient screening tool based on situational needs, each performing similarly well.

This study is not without weaknesses worthy of notice. First, PDEQ assessments in this work were taken at a median of 9 days from the traumatic event and not during ED treatment. However, the finding that the timing of PDEQ measurement (within the 0 to 30 day time bracket) did not modify the instrument’s performance as a risk estimator reduces the likelihood of a major difference in predictive accuracy between ED and subsequent assessment timing.

Second, this work considered one of several metrics of peritraumatic reactions, the PDEQ, and thereby might have preferentially captured the effect of peritraumatic dissociation (i.e., derealization and depersonalization) rather than other immediate reactions to trauma exposure. Indeed, previous work suggests that peritraumatic dissociation and peritraumatic distress (as captured by the PDI) independently contribute to downstream PTSD [22]. Having tested the PDEQ alone, we relate to this work as a *“proof of concept”* study. The extent to which other peritraumatic measurements can be similarly employed is yet to be determined.

A third apparent limitation is that, once the covariates are included in the predictive model, the predicted probabilities are characterized by wide confidence intervals in some cases, thereby at times reducing the model’s precision. However, as can be seen in Table 4, the predicted probabilities are significantly influenced by covariates such as sex, mechanism of injury, and previous trauma exposure. For example, a PDEQ score of 20 can result in predicted probabilities ranging from 5.29 (CI = [1.95, 13.6]) for a male with current non-interpersonal trauma and no history prior trauma exposure, to 57.29 (CI = 36.11, 76.11) for a female with current interpersonal trauma and prior history interpersonal trauma exposure, and non-overlapping CIs. Thus, while the model employing the PDEQ alone has more precision (i.e., less variance) than the model that included covariates, its predictions are, importantly, biased by ignoring their effects. As such, while potentially decreasing the precision of the model, the covariates examined here reduce the prediction bias of the PDEQ-only model, and provided critical information about moderators of acute PTSD risk estimates upon ED admission. Lastly, the calibration slope indicates that the PDEQ alone model is slightly under-fitted. This number suggests that while the improvements in Brier Skill Score and AUC are modest in the adjusted model, the model is also better able to capture the underlying structure of the data than the PDEQ alone model.

Fourth, PTSD diagnoses in this study were based on DSM-IV diagnostic criteria rather than the currently used DSM-5. The extent to which the predictive model performs equally well in predicting DSM-5 acute PTSD is an inquiry worthy of scientific rigor.

Lastly, several potential confounders of PDEQ prediction, such as race, income, and education, as well as other known PTSD risk indicators, such as ED heart rate [39], head injury [40], general distress, or ED pain levels [37] were not included in the work, and their eventual effects remain untested.

Our results require replications and external validations to be safely generalized. However, in this work, the stacked model and the stratified model performed equally well across participating studies, providing preliminary evidence that the generic, stacked model used in this study could be applied to diverse acute care settings.

## Conclusion

This work suggests that instruments measuring an initial reaction to trauma exposure, i.e., peritraumatic and ED distress, could be used to screen individuals in need of subsequent assessment of emerging and properly termed *post-traumatic* symptoms that are highly predictive of longer-term PTSD. It thereby informs the rationale and suggests the potential cost-effectiveness of staged, sequential assessment of PTSD risk in recent survivors seen in acute care centers.

## Data Availability

The datasets analyzed during the current study are available from the corresponding author upon reasonable request. The risk likelihood estimates calculator is freely available at https://wvdmei.shinyapps.io/results_lookup/

## Abbreviations

AUC: Area under the receiver-operating characteristic curve
CAPS: Clinician-administered PTSD scale
CI: Confidence interval
DSM: Diagnostic and statistical manual of mental disorders
ED: Emergency department
ICPP: International Consortium to Predict PTSD
MVA: Motor vehicle accident
OR: Odds ratio
PDEQ: Peritraumatic dissociative experiences questionnaire
PDI: Peritraumatic distress inventory
PTSD: Post-traumatic stress disorder
SE: Standard error
