# Correction to: Evaluating a screener to quantify PTSD risk using emergency care information: a proof of concept study

**DOI:** 10.1186/s12873-020-00346-7

**Published:** 2020-06-29

**Authors:** Willem F. van der Mei, Anna C. Barbano, Andrew Ratanatharathorn, Richard A. Bryant, Douglas L. Delahanty, Terri A. deRoon-Cassini, Betty S. Lai, Sarah R. Lowe, Yutaka J. Matsuoka, Miranda Olff, Wei Qi, Ulrich Schnyder, Soraya Seedat, Ronald C. Kessler, Karestan C. Koenen, Arieh Y. Shalev, International Consortium to Predict PTSD, Yael Errera-Ankri, Sarah Freedman, Jessie Frijling, J. Carel Goslings, Jan Luitse, Alexander McFarlane, Derrick Silove, Hanspeter Moergeli, Joanne Mouthaan, Daisuke Nishi, Meaghan O’Donnell, Mark Rusch, Marit Sijbrandij, Sharain Suliman, Mirjam van Zuiden

**Affiliations:** 1 Department of Population Health, New York University Langone Health, 227 E 30th St, New York, NY USA; 2 Department of Psychiatry, New York University School of Medicine, 1 Park Avenue, New York, NY 10016 USA; 3 Department of Epidemiology, Columbia University Mailman School of Public Health, 722 W. 168th St, New York, NY 10032 USA; 4 School of Psychology, University of New South Wales, Sydney, NSW 2052 Australia; 5 Department of Psychological Sciences, Kent State University, 144 Kent Hall, Kent, OH 44242 USA; 6 Department of Surgery, Medical College of Wisconsin, 9200 W. Wisconsin Avenue, Milwaukee, WI 53226 USA; 7 Department of Counselling, Developmental, and Educational Psychology, Lynch School of Education and Human Development, Boston College, Campion Hall Room 313, 140 Commonwealth Avenue, Chestnut Hill, MA 02467 USA; 8 Department of Psychology, Montclair State University, 1 Normal Avenue, Montclair, NJ 07043 USA; 9 Division of Health Care Research, Center for Public Health Sciences, National Cancer Center Japan, 5-1-1 Tsukiji, Chou-ku, Tokyo, 104-0045 Japan; 10 Department of Psychiatry, Academic Medical Center, University of Amsterdam, Meibergdreef 9, 1105 AZ Amsterdam, The Netherlands; 11 Arq Psychotrauma Expert Group, Postbus 240, 1110 AE Diemen, The Netherlands; 12 Department of Psychiatry and Psychotherapy, University Hospital Zurich, Box 1931, Lenggstrasse 31, 8032 Zürich, PO Switzerland; 13 Department of Psychiatry, Stellenbosch University, Private Bag X1, Matieland, Stellenbosch, 7602 South Africa; 14 Department of Health Care Policy, Harvard Medical School, 180 Longwood Avenue, Boston, MA 02115 USA; 15 Department of Epidemiology, Harvard T.H. Chan School of Public Health, Kresge 505, 677 Huntington Avenue, Kresge Building, Boston, MA 02115 USA

**Correction to: BMC Emerg Med 20, 16 (2020)**

**https://doi.org/10.1186/s12873-020-00308-z**

The original article [1] contains an error in the presentation of institutional author , J Carel Goslings’s name in the Acknowledgements sub-section which is presented correctly in this Correction article.

**Acknowledgements**

Members of the ICPP also include Yael Errera-Ankri, Sarah Freedman, Jessie Frijling, J Carel Goslings, Jan Luitse, Alexander McFarlane, Derrick Silove, Hanspeter Moergeli, Joanne Mouthaan, Daisuke Nishi, Meaghan O’Donnell, Mark Rusch, Marit Sijbrandij, Sharain Suliman, and Mirjam van Zuiden. We are grateful for the contributions of Paul O’Connor at the Nathan Kline Institute for his invaluable assistance in data management and quality assurance.
